# Supplementary material for: Effects of Maternal Supplementation with Rare Earth Elements during Late Gestation and Lactation on Performances, Health, and Fecal Microbiota of the Sows and Their Offspring
Source: Animals (Basel). 2019 Sep 28;9(10):738. doi: 10.3390/ani9100738 (PMC6826669; doi:10.3390/ani9100738)
Supplement: Supplementary file 1 [file animals-09-00738-s001.pdf]

Effects of Maternal Supplementation with Rare Earth Elements during Late Gestation and Lactation on Performances, Health, and Fecal Microbiota of the Sows and Their Offspring

Yi Xiong, Jiaman Pang, Liangkang Lv, Yujun Wu, Na Li, Shimeng Huang, Zhi Feng, Ying Ren and Junjun Wang

A

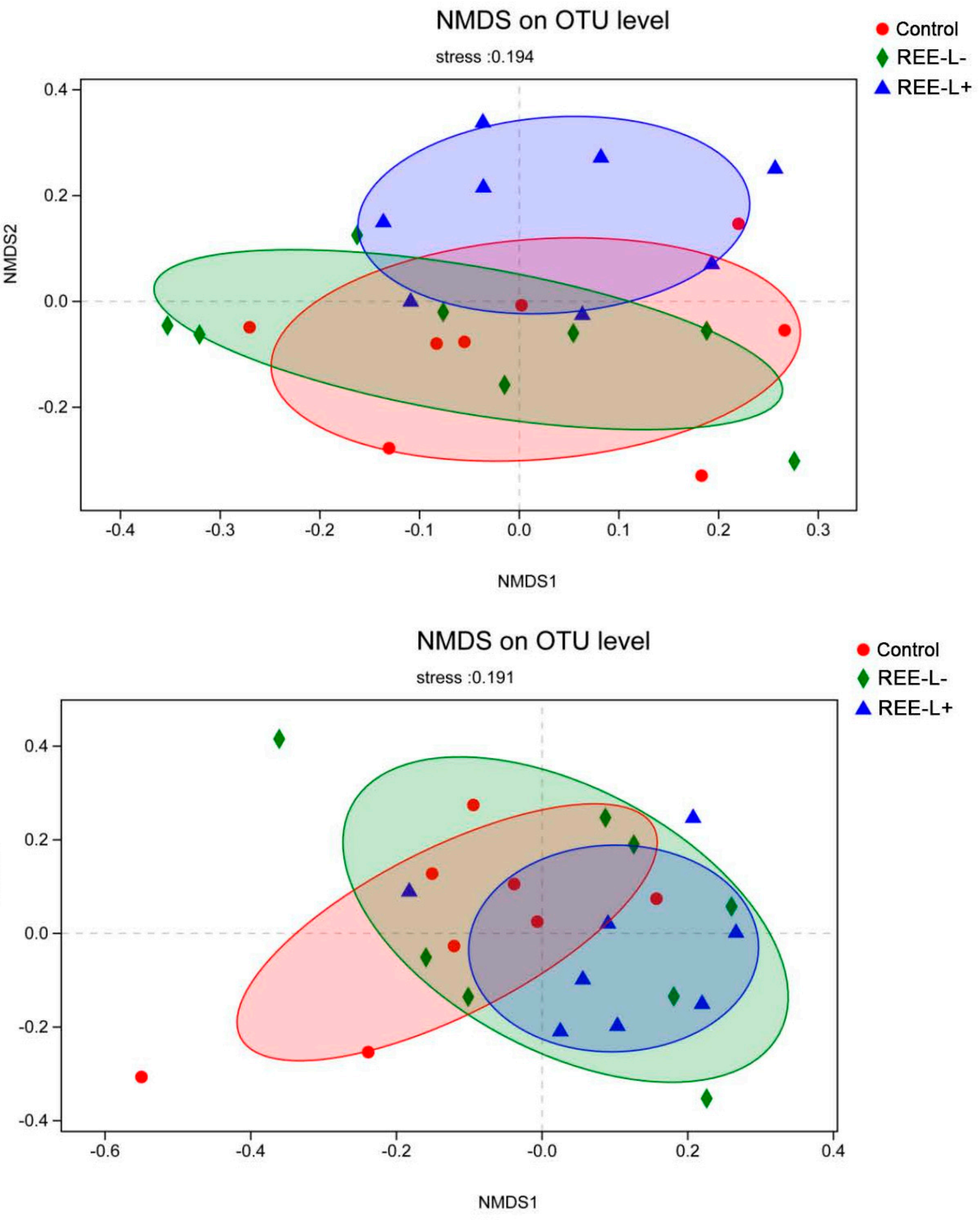

B

Figure S1. Non-metric multidimensional scaling analysis (NMDS, Bray–Curtis distance) plot of (A) the sows and (B) their piglets. n = 8 per group.

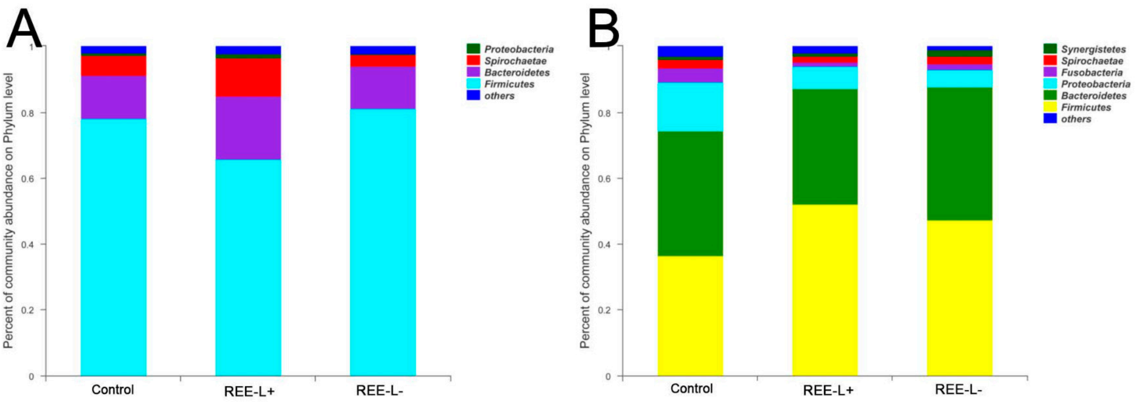

Figure S2. Abundant phylum in fecal microbiota of (A) the sows and (B) their piglets. n = 8 per group.
